# The role of urban forest patches in maintaining isopod diversity (Oniscidea)

**DOI:** 10.3897/zookeys.801.22829

**Published:** 2018-12-03

**Authors:** Elisabeth Hornung, Andrea Kásler, Zsolt Tóth

**Affiliations:** 1 Department of Ecology, Institute for Biology, University of Veterinary Medicine Budapest, H-1077 Budapest, Rottenbiller str. 50, Hungary University of Veterinary Medicine Budapest Budapest Hungary

**Keywords:** biotic homogenisation, disturbance tolerance, ecological character, habitat specialist, urbanisation index, woodlice

## Abstract

Compositional changes in natural communities associated with anthropogenic influence often lead to localised extinctions and biodiversity loss. Soil invertebrates are also threatened by urbanisation due to habitat fragmentation, vegetation changes and management, soil alteration, degradation, and disappearing shelter sites. The aim was to assess terrestrial isopod (Oniscidea) assemblages in differently degraded urban forest patches of a metropolitan area (Budapest, Hungary). Study sites were compared by their species richness, composition and the relevant background factors (soil properties, dead wood, litter characteristics, and canopy closure). The degree of urban disturbance was expressed using an urbanisation index (UI) based on built-up density and vegetation cover. The isopods were identified to species level, and were qualified by their habitat preference and naturalness index (TINI). Average Rarity Index (ARI), derived from TINIs provided information on the degree of naturalness/disturbance of each habitat. Altogether 14 isopod species were collected from 23 sample sites. Urbanisation indirectly affected on the composition of isopod assemblages through the quantity of dead wood and soil plasticity. ARIs and UIs of sample sites were negatively correlated. Urban patches harboured habitat generalist, synanthropic and established introduced species with low naturalness value of assemblages. Areas with no or low anthropogenic disturbance maintained stable native, autochthonous assemblages that were characteristic of rural sites in the region. Transitional zones between rural and urban habitats usually maintained a mixed isopod fauna consisting of both urban and rural elements.

## Introduction

Currently increasing number of studies explore the effects of urbanisation on biological communities at a global level (Niemelä et al. 2000, McKinney 2008, Richter and Weiland 2012, Wang et al. 2012a). The alteration and fragmentation of natural habitats generally leads to a shift in species composition, resulting in biotic homogenisation and changes in ecosystem services as well (McPherson 1998, Whitford et al. 2001, McKinney 2006, Tratalos et al. 2007). Human activity, such as construction industry, air pollution and pollutant emissions of vehicles and the use of chemicals, contributes to urban soil degradation (Pouyat et al. 2008).

The majority of soil invertebrates are highly sensitive to disturbances (Barbercheck et al. 2009) and environmental changes (Santorufo et al. 2012). This includes the macrodetritivore fauna, which has an important role in the ecosystems’ nutrient cycling. These invertebrates fragment dead plant material through their feeding activity increasing its surface area and promoting microbial decomposition (Hanlon and Anderson 1980, Bardgett 2005). Woodlice (Isopoda: Oniscidea) are one of the major invertebrate group contributing to these processes (Anderson 1988, Paoletti and Hassall 1999).

Terrestrial isopods can be used as ecological indicators of habitat qualification. They are widespread, limited in their dispersal abilities, and relatively easy to collect and identify. Based on a single species’ ecological needs and tolerances, species composition informs us about habitat characteristics including habitat disturbance/naturalness (Vilisics et al. 2007, Tuf and Tufová 2008, Hornung et al. 2008, 2009). Urban areas are hot spots for species introduction (Vilisics and Hornung 2009) threatening natural communities. Successfully established introduced species are usually eurytopic and/or cosmopolitan ones leading to global urban soil fauna homogenisation and convergence (McKinney 2006, Pouyat et al. 2015).

To study the effects of urbanisation on oniscidean fauna differently urbanised woodland habitat patches were compared. We expected that our data would be in accordance with the following hypotheses:

(1) the ’Intermediate Disturbance Hypothesis’ (IDH; Connell 1978) that predicts diversity being the highest in habitat with moderate levels of disturbance;

(2) the ’Habitat specialist hypothesis’ that indicates that the abundance and species richness of forest specialist species will decline along a rural–suburban–urban gradient (Magura et al. 2008) and

(3) the ’Synanthropic species hypothesis’ that predicts that abundance and species richness of synanthropic species will increase along a rural–suburban–urban gradient (Magura et al. 2008).

## Materials and methods

### Study sites and design

**Figure 1.**
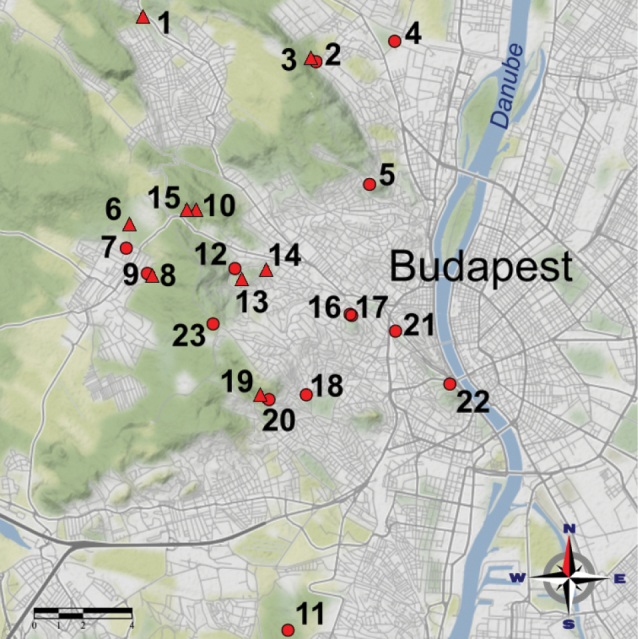
The arrangement of sample sites (23) in Buda, the western, hilly side of Budapest. The numbers indicate the sample sites (see Suppl. material 1). Symbols indicate rural (▲) and disturbed (●) habitats.

The Budapest metropolitan area is divided by the Danube River, which separates the two major parts of the city, Buda and Pest. Buda can be characterised by a uniform parent rock (primarily limestone and dolomite). The area included in this study is in the urbanised area of the Buda Hills. Historically, Buda was covered by continuous natural forest that was fragmented by the growing city. Rural and differently degraded urban forests and other woody patches (e.g. planted forests, parks, gardens, and cemeteries) were selected in Buda (Fig. 1). Rural forests, situated in the Buda Landscape-Protection Area, represented semi-natural woodlands.

### Landscape structure characteristics

To quantify urbanisation intensity an urbanisation index (UI) was applied as proposed in Seress et al. (2014). Vegetation cover, building density, and the presence of sealed surface (roads) were scored for 100 cells of 1 km^2^ area around each study site using the QGIS software (version: 2.16). For each site, the urbanisation index was calculated by extracting the first principal component (PCA1) from a principal component analysis (PCA) of five urbanisation variables (mean building density, number of cells with high building density, number of cells with road, mean vegetation density, number of cells with high vegetation density).

### Soil sampling and analyses

Composite samples were taken from 0–15 cm of the topsoil layer of each study site. Soil physicochemical properties were determined at the Soil Conservation Laboratory of National Food Chain Safety Office (Velence, Hungary). Soil pH (H_2_O) was measured in 1:2.5 soil:water suspensions for 12 h after mixing. Soil organic matter (SOM, m/m %) was determined by the standard ignition method. Total soluble salt content of the soil (m/m %) was measured with a conductometre (Radelkis OK-102/1). To characterise soil texture, the soil plasticity index (K_A_) that refers to the soil clay content, was applied (MSZ-08-0205 1978). Soil CaCO_3_ (m/m %) was determined with a Labor MIM calcimetre (MSZ-08-0206-2 1978).

### Vegetation characteristics

Structural attributes of vegetation important for isopods were recorded using a 10 × 10 m quadrat at each site, in May and October, 2016. Percentage cover of dead wood and litter, canopy closure was estimated visually (Jennings et al. 1999, Humphrey and Bailey 2012), while litter depth was measured with a ruler. For data analysis, variables were classified into the following categories:

- amount of dead wood: 1 (0 %), 2 (0–20 %), 3 (> 20 %)

- litter cover: 1 (0–35 %), 2 (36–65 %), 3 (66–100 %)

- canopy closure: 1 (0–35 %), 2 (36–65 %), 3 (66–100 %)

- litter depth: 1 (0 cm), 2 (0–1.5 cm), 3 (> 1.5 cm)

### Isopod sampling and species/habitat qualification

Terrestrial isopods were collected by time-restricted hand sorting (60 minutes per site) during their main activity seasons, in May and October, 2016. To ensure that rare or habitat specialist species were not missed, special attention was paid to favourable microhabitats, such as leaf litter, fallen tree trunks or branches, and shelter sites under bark and stones. Individuals were preserved in 70 % ethanol and later identified to species level using the key by Gruner (1966). Species nomenclature follows Schmalfuss (2003).

Isopod species were categorised using the terminology by Williamson and Fitter (1996). Introduced species with a self-sustaining population were called ‘established introduced’. Species distributed in most continents were considered ‘cosmopolitans’. Synanthropic label was applied for species that are connected with built-up areas and do not occur in the wild. Non-native species were those that went through adaptation and occur in suburban – semi-natural fringe areas. Native species are autochthonous and/or have been established by dispersal/dispersion presumably before historical times.

In species qualification we utilised the Terrestrial Isopod Naturalness Index (TINI) under development. This additive index is based on the following attributes of the single woodlouse species: global (cosmopolitan – endemic), regional (frequent – rare) distribution, ecological (habitat generalist – specialist) and disturbance tolerance (Hornung et al. 2007a, 2008, 2009). In this ranking system introduced and/or common species are assigned 0 or low while endemic and/or rare species receive high scores (max. 20). Species found in this study were ranked based on the nomenclature of Hornung et al. (2007a, 2008).

The ecological tolerance of woodlice sets the limits of their occurrence. Considering their poor dispersal abilities (philopatry) and the ecological features (TINI) of the species present at a location makes them available for the characterisation of the habitats in question: species composition reflects habitat quality. By applying TINI scores of species to an assemblage one can compile scores assigned to the habitat. The summed TINI indices of species standardised by the number of species present gave Average Rarity Index (ARI = ∑TINI/N where N is the number of species in the assemblage). This index results in a novel way to compare different localities involving a single species’ naturalness – disturbance tolerance. This way it gives a more realistic, qualitative biodiversity indicator than simply species richness.

### Statistical analyses

All statistical analyses were performed in R software version 3.2.5., using the R packages ‘lme4’ (Bates et al. 2015) and ‘mvabund’ (Wang et al. 2012b). Hierarchical cluster analysis was carried out with the software PAST 3.10 (Hammer et al. 2001). Relationships between species richness, composition of isopods and environmental variables (soil properties, vegetation characteristics, and urbanisation index) were tested by generalised linear mixed models (GLMMs). After fitting the full models for each dependent variable, Akaike Information Criterion (AIC) was used to select the most parsimonious model. For species composition analyses, GLMMs with a multivariate approach were applied. Since we had presence-absence data, the ‘manyglm’ method (family = binomial) was used. The sampling time (spring, autumn) was considered as a random factor in the models. To compare isopod assemblages of study sites, a hierarchical cluster analysis with the Jaccard similarity index was carried out. The myrmecophilous *Platyarthrushoffmannseggii* was excluded from this analysis because its accidental occurrence connected to ant nests. Spearman rank correlation tests were performed to examine relationships between soil properties, ARI and UI. Assumptions of normality and homoscedasticity of the residuals were verified visually using diagnostic plots. Statistical significance was determined at the level: α = 0.05.

## Results

### Urbanisation intensity of sample sites

**Figure 2.**
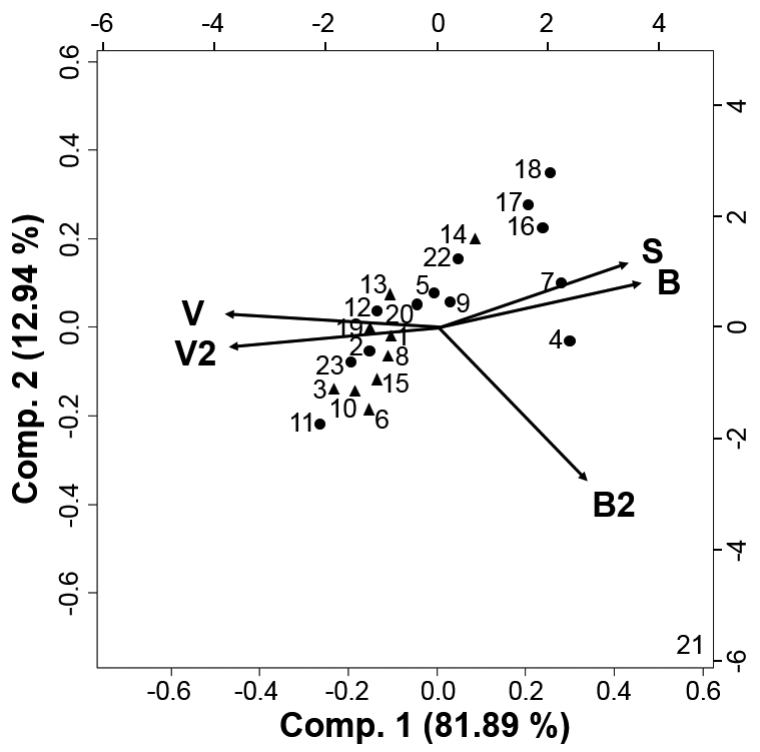
PCA biplot of the sample sites according to the urbanisation variables. Abbreviations: B - mean building density, B2 - number of cells with high building density, S: number of cells with road, V: mean vegetation density, V2: number of cells with high vegetation density. Numbers are sample site (same as in Fig. 1). Symbols indicate rural (▲) and disturbed (●) habitats.

The 23 sampling locations in this study had the same parent material, but differed in the amount of woody vegetation cover that varied from planted trees through isolated forest remnants to rural forests. Sampling plots represented differently urbanised habitat fragments. Intensity of anthropogenic disturbance was expressed in urbanisation indices (UI). Urbanisation indices ranged from -2.58 to 5.56 (Suppl. material 1) with higher values indicating more urbanised habitats. According to the results of PCA, sample sites were arranged along a gradient that reflects the intensity of urbanisation (Fig. 2). The pattern showed a more or less continuous transition from rural to the most disturbed habitats. The first two principal components explained 94.83 % of the total variance of the dataset.

### Relationship between soil, vegetation characteristics, and urbanisation intensity

**Figure 3.**
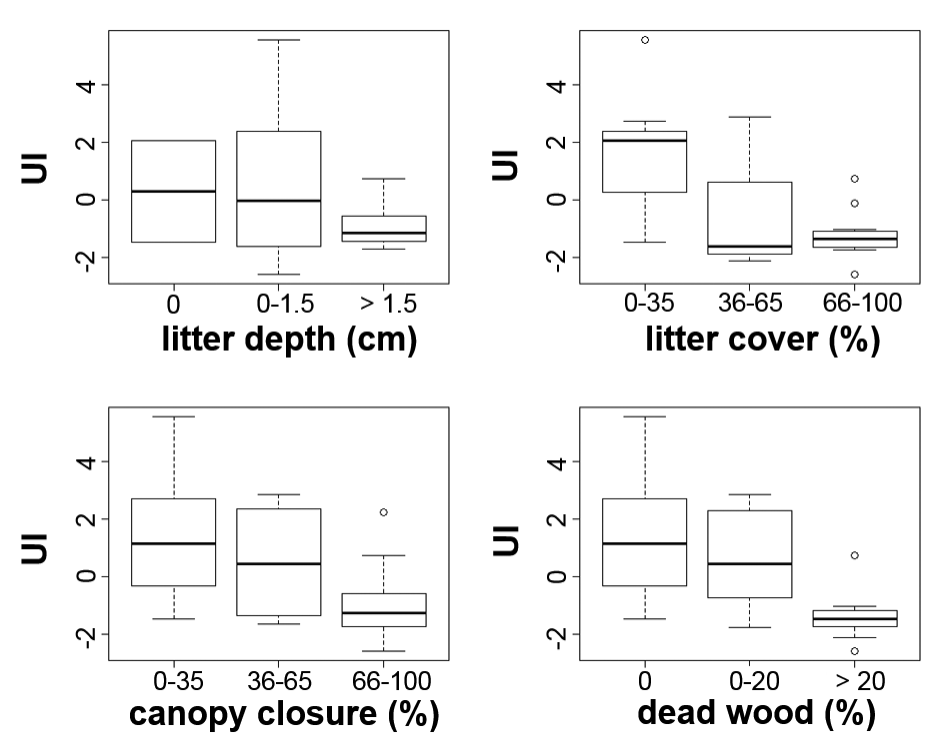
Box plots of urbanisation intensity (UI) according to vegetation characteristics. Horizontal line within a box indicates the median.

The soils of the sample sites did not show high variability, with the exception of the CaCO_3_ content (Suppl. material 1), which had a positive correlation with the urbanisation index (r_s_ = 0.377, p = 0.001). In contrast, SOM content (r_s_ = -0.363, p = 0.013) and soil plasticity (K_A_) (r_s_ = -0.321, p = 0.029) were negatively influenced by urbanisation intensity. Soil pH and total soluble salt content showed marginally significant positive relationships (r_s_ = 0.256, p = 0.086 and r_s_ = 0.250, p = 0.095 respectively) with urbanisation. More natural habitats (with lower UIs) had higher litter depth and cover, canopy closure and amount of dead wood compared to the more urbanised ones (Fig. 3).

### Isopod diversity and species scores

Altogether 14 isopod species were recorded during the survey (Table 1). Eight of them were native, but differed in habitat preference and frequency of occurrence. Four species were categorised as cosmopolitan, one as established introduced, and three were synanthropic (Table 1). *Armadillidiumvulgare*, *Orthometoponplanum*, and *Protracheoniscuspolitus* were the most widespread ones among the sampled spots. The distribution of *A.vulgare* differed from that of the other two species: the presence of the latter two indicated low or no human disturbance (low or negative UI indices, high ARI values of sampling localities). *Armadillidiumvulgare* showed up as a constant element of urbanised neighbourhood, sometimes being the only representative of the isopod fauna (site 11). It occurred in 13 out of the 23 sampled habitats. These plots proved to be highly urbanised, or in some cases transitional areas. No rare (either native rare or disturbed rare) species were found.

**Table 1.** The collected Oniscidea species, their naturalness scores (TINI), and frequency of occurrence. (Categories are given according to Hornung et al. 2008 and are valid for the Pannonian region).

Family	Species	Species category	TINI	Number of sites of occurrence
** Agnaridae **	*Orthometoponplanum* (Budde-Lund, 1885)	native frequent	19	11
	*Protracheoniscuspolitus* (C. L. Koch, 1841)	native frequent	17	9
** Armadillidiidae **	*Armadillidiumvulgare* (Latreille, 1804)	cosmopolitan, widely distributed	9	13
** Cylisticidae **	*Cylisticusconvexus* (De Geer, 1778)	established introduced	10	3
** Platyarthridae **	*Platyarthrushoffmannseggii* Brandt, 1833	cosmopolitan widely distributed	12	5
** Porcellionidae **	*Porcellioscaber* Latreille,1804	cosmopolitan synanthropic	3	8
	*Porcelliospinicornis* Say, 1818	established introduced synanthropic	8	3
	*Porcellionidespruinosus* (Brandt, 1833)	cosmopolitan synanthropic	7	3
** Trachelipodidae **	*Porcelliumcollicola* (Verhoeff, 1907)	native widely distributed	10	4
	*Trachelipusnodulosus* (C. L. Koch, 1838)	native widely distributed	11	1
** Trichoniscidae **	*Androniscusroseus* (C. L. Koch, 1838)	native	13	1
	*Haplophthalmusmengii* (Zaddach, 1844)	native	13	1
	*Hyloniscusriparius* (C. L. Koch, 1838)	native widely distributed	10	4
	*Trichoniscuspusillusagg* Brandt, 1833	native widely distributed	12	3

The native species (*O.planum*, *Pr.politus*, *Porcelliumcollicola*, *Androniscusroseus*, *Haplophthalmusmengii*, *Hyloniscusriparius*, and *Trichoniscuspusillus*) were restricted to rural and/or fringe areas. Cosmopolitan, introduced established and synanthropic species included *A.vulgare*, *Cylisticusconvexus*, *Porcellioscaber*, *P.spinicornis*, and *Porcellionidespruinosus* and occurred in disturbed, human dominated places. The typical urban isopod assemblage consisted of *A.vulgare*, *P.scaber*, *Ps.pruinosus* (in order of prevalence), and occasionally *P.spinicornis* and *C.convexus*. These species were not found in rural woodlands.

Widely distributed, hygrophilic, mainly endogeic species were: *A.roseus*, *H.mengii*, and *Hy.riparius* (sites 2, 4, 16, 18 and 23). A highly managed urban park (site 21) and green verges along pavements (sites 7, 9) still harbour one or two woodlouse species, most often *A.vulgare* and/or *P.scaber*. Fringe areas, that is, rural – urban transition zones or ecotones (sites 2, 4, 14, 16, 17, and 18) had a mixed isopod fauna, resulting the highest species richness (Suppl. material 2). High species dissimilarity (85–100%) occurred within ten metres (sites 2, 3 and 8, 9) in localities where built-in area sharply turns into quasi-natural forest.

**Figure 4.**
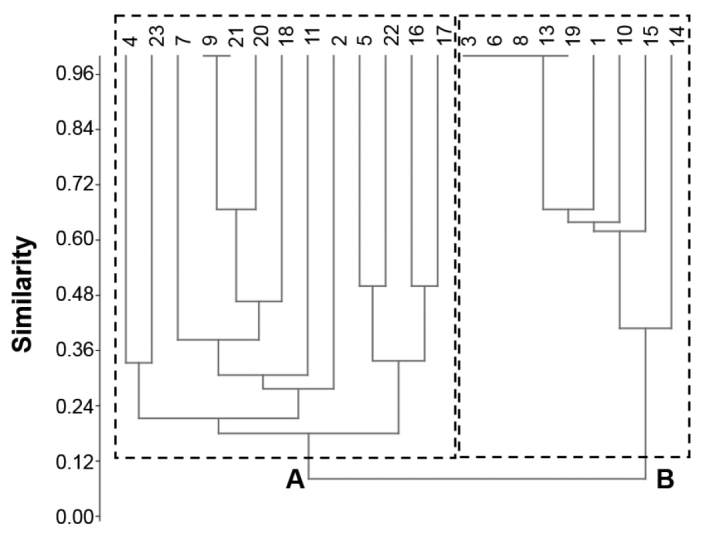
Hierarchical cluster analysis dendrogram showing two main groups (A and B) based on the species composition of isopod assemblages. Numbers on the top are the numbers (ID-s) of the sample sites (for IDs see Suppl. material 1).

The hierarchical cluster analysis clearly separates the isopod assemblages into two groups (Fig. 4). Group ’A’ consists of urbanised or transitional habitats dominated by the previously mentioned urban species assemblage, while group ’B’ refers to rural areas predominated by the presence of *O.planum* and *Pr.politus*. Site 14 seemed to be separated within group B.

### Effects of habitat characteristics on woodlice assemblages

According to the results of the GLMMs, the quantity of dead wood (Dev = 108.27, p < 0.001) and soil plasticity (Dev = 49.36, p = 0.002) were the most significant environmental variables affecting species composition. *Orthometoponplanum* and *Pr.politus* (native species with high TINIs) preferred sample sites with high amount of dead wood (Dev = 13.88, p = 0.005 and Dev = 23.23, p = 0.001, respectively), while *P.scaber*, a cosmopolitan species with low TINI showed the opposite trend (Dev = 22.03, p = 0.001). The presence of *A.vulgare*, a cosmopolitan species with medium TINI, was primarily determined by soil texture (Dev = 8.49, p = 0.05): it was mainly found in habitats where soil clay content (expressed in K_A_) was low. While individual species exhibited preference towards certain habitat parameters, total species richness of isopods was not affected.

### Relationship between Average Rarity Index (ARI) and urbanisation intensity (UI)

**Figure 5.**
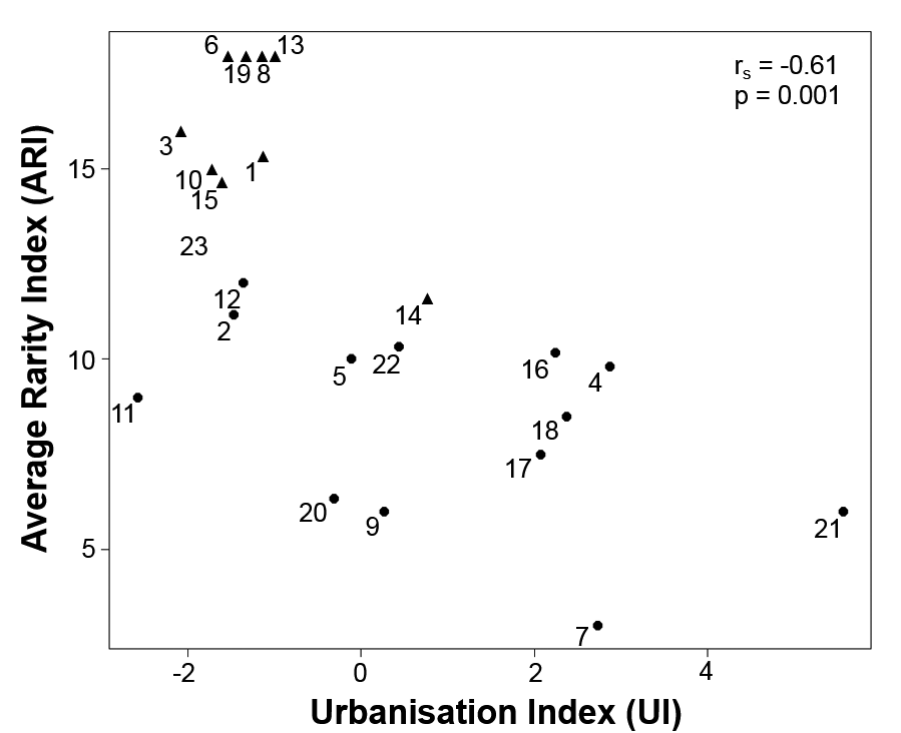
Average Rarity Index (ARI) decreased with higher urbanisation (UI). Higher value of UI means increasing urbanisation. Numbers are habitat identifiers, see Fig. 1 and Suppl. material 1 (▲ – rural, ● – differently disturbed habitats).

The Spearman rank correlation test showed negative correlation between ARI based on species qualification of isopods and UI (Fig. 5). The ARI values significantly decreased with the urbanisation level of habitats. Rural, semi-natural habitats form a group (see ▲ symbols on Fig. 5) with the highest ARI scores (13 – 18) and low UIs (-2.11 – -1.03, except site 14 with UI = 0.74). ARIs of urban and fringe habitats are under these values (6 – 13), while UI scores of this group are highly variable (-2.58 – +5.56).

## Discussion

Urban soils are overwhelmed by strong human physical effects (e.g. grading and irrigation) and tend to lack the effects of native factors (e.g. topography and drainage) that formed development of soil characteristics during a long time period. These soils vary widely in their characteristics and are dependent on both direct and indirect effects resulting from urban land use and cover change (Pouyat et al. 2010). The various plots differed slightly in soil parameters, mainly in CaCO_3_ content. In accordance with results of previous urban studies (Craul and Klein 1980, Short et al. 1986) higher CaCO_3_ content, higher pH, but lower humus content and soil plasticity (K_A_) was experienced in habitats with high urbanisation index compared to rural sites. Building materials, for example concrete, asphalt and bricks can be the sources of increased CaCO_3_ level (Alexandrovskaya and Alexandrovskiy 2000), which can lead to higher soil pH (Pouyat et al. 2015).

The 14 isopod species found in our survey represent 50% and 25% of the recorded fauna of Budapest and Hungary, respectively (Hornung et al. 2007a, 2008, Vilisics and Hornung 2008, 2009). Korsós et al. (2002) and later Vilisics and Hornung (2009) summarised data on the oniscidean fauna of the entire metropolitan area of Budapest, including different kinds of man-made habitats, e.g. gardens, courtyards, densely built-up areas and botanical gardens. The present study focused only on a subset of the diverse urban habitat types on the Buda side of the city and thus fewer species were detected.

Although average species richness (α diversity) is usually low in Hungary, three species per location on average (Hornung et al. 2008), in the present study 6 locations out of the 23 (26%) resulted 4–6 species. These habitats could be referred to fringe areas. The species number and assemblage composition of some other European cities are also known. In an urban study of Olomouc, Czech Republic, sampling city parks, built up areas, gardens, ruderal and natural habitats altogether 17 woodlice species were collected (Riedel et al. 2007). In relation to habitat naturalness the species spectrum was dominated by adaptable and eurytopic species (sensu Tuf and Tufova 2008) meaning habitat generalist species. Ferenți et al. (2015) published eleven species from 19 localities within Salonta town (western Romania) with the highest (6) species richness from a wetland habitat and most sampling sites harbouring only one or two species. In Bucharest (Romania) 17 species were sampled by Giurginca et al. (2017). In three Swiss cities 17 species were mentioned by Vilisics et al. (2012). The highest species richness was found in Lucerne (13), while Zurich had eleven and Lugano nine species. The data mentioned above are influenced by difference in sampling methods, but give a tentative picture of the species richness of different geographic regions. The natural species pools surrounding urban areas in the different (bio)geographical areas may also differ within Europe. In addition to the basic European fauna they may contain different regional biogeographical elements.

The results of the present study failed to fulfil the requirements of the Intermediate Disturbance Hypothesis. There was no consistent pattern in species richness distribution. However, urban habitats harboured more species on average, but without any statistically significant correlation. In a rural – urban gradient study (Debrecen, Eastern Hungary) IDH was also not proved for woodlice, while it was valid for millipedes (Hornung et al. 2007b, Bogyó et al. 2015). In another extensive urban study (Pécs/Hungary; Farkas and Vilisics 2006) either habitat generalists (*A.vulgare*, *Po.collicola*, *Trachelipusrathkii*) or synanthropic species (*P.scaber*, *C.convexus*, *Ps.pruinosus*) dominated densely built-in areas while in the city edge ecotone zones the more habitat specialists also joined the assemblages (*T.nodulosus* and *Pr.politus*). Rural – urban transition zones and ecotones also had a mixed isopod fauna in the present study.

The Habitat specialist and the Synanthropic species hypotheses (Magura et al. 2008) were confirmed by the present study. We experienced a species exchange between habitat specialists and generalists and/or synanthropic species along the rural – urban gradient. Typical forest species (*Pr.politus*, *O.planum*) were constant elements of rural habitats while habitat generalist and/or synanthropic species (porcellionids, *A vulgare*, *C.convexus*) constituted the species assemblages of urbanised forest patches.

The apparent negative association of *P.scaber*, a typical urban faunal element, with dead wood can be explained by its high tendency for aggregation (Broly et al. 2012). Hornung et al. (2008) found that species in the family Porcellionidae show a clear preference for human settlements in Hungary. *Porcellioscaber*, *C.convexus* and *Ps.pruinosus* are typical elements of urban ecosystems and farmlands. They are common also in many other parts of the world (Hornung et al. 2008). The unusual appearance of species with a low desiccation tolerance (Trichoniscidae species with hygrophilic and/or endogeic nature) was always connected with some kind of constant water supply, e.g. public artesian wells (sites 4, 18, 16). The significant correlation of *O.planum* and *Pr.politus* with the amount of wood debris can be attributed to their typical sylvicolic nature (Hornung et al. 2008, Tomescu et al. 2008). Geographical distance may also be important. The inner city is fairly isolated from rural areas preventing species dispersal and colonisation.

Isopods are indicators of the naturalness of vegetation, and the quality and quantity of dead wood and litter in their habitats, which are used by them both as food and shelter (Rushton and Hassall 1983). Habitats with a lot of dead wood were favourable for many natural species. Dead wood was the main driver of species composition in isopod assemblages, probably because they largely contribute to the supply of organic matter and affect microclimatic variables, e.g. humidity. Preventing removal of dead wood from urban green spaces could enhance the survival of the epigeic invertebrate fauna (Bang and Faeth 2011). This indicates the importance of small-scale management decisions on local biodiversity. Urban planning and changes in the management of public areas, e.g. retaining leaf litter, might be advantageous for soil fauna survival which in turn provide food for various vertebrates and may increase biodiversity as a whole (Stagoll et al. 2010, Threlfall et al. 2016).

However, the 1 × 1 km area units used for determination of UIs seems to be a too broad a scale compared to the small-scale heteromorphic sensibility of the investigated flightless epigeic macrodetritivore fauna. Their habitat preference may be controlled on a much finer scale (see also McCary et al. 2017). UI might be also misleading in some cases as e.g. forests with high hiking traffic but without any buildings and sealed surfaces (sites 11 and 23 in our case). These showed low urbanisation (negative UI scores) and naturalness values (ARI) but impact of anthropogenic disturbance is hidden. Similarly, sharp habitat boundaries with mixtures of urban and rural species explains the exceptional position of sample site 14 on Figs 4 and 5. The UIs and the total naturalness index of isopod species (∑TINI) at the sample sites did not show significant relatedness, but the habitats’ ARIs did, which confirms previous statements (Hornung et al. 2009): ARI is a good tool in estimating habitats’ naturalness as it involves the species’ ecological features and enables a more refined evaluation. High species number does not always mean high naturalness from a conservation biological point of view.

Urbanisation often leads to changes in species richness and community composition. New landscapes and habitats are formed that do not occur elsewhere (Niemelä 1999, Mabelis 2005). Species have different responses to anthropogenic habitat modification, depending on their ecological needs and tolerance. Urbanisation worldwide is accompanied by the occurrence and dominance of habitat generalist species with broad tolerances and the establishment of introduced, mainly synanthropic species. These changes lead to homogenisation and convergence of urban faunas on both local and global scales.

## Conclusions

Woody habitats within the urban matrix can still support biodiversity to varying degrees. As species have different responses to anthropogenic impacts, the species composition of urban areas can depend greatly on the habitat characteristics of the local and surrounding areas and their distances from natural species pools. Urban patches harbour assemblages that are relatively modest in species richness and have low naturalness values. The composition usually consists of typical homogenising urban species. Transitional zones (fringe areas) between rural and urban habitats might maintain an assemblage of rural, habitat specialist elements with high naturalness value mixed with urban ones. Areas with no or low disturbance maintain species poor but stable native, autochthonous assemblages, with high naturalness value, characteristic for rural sites in the region.

The Terrestrial Isopod Naturalness Index (TINI) and the Average Rarity Index (ARI) give good possibilities to assess urban effects on habitats and serve as potential tools for habitat qualification. Our study demonstrates that maintaining litter layer with dead wood in urban habitats is an essential factor for favouring natural/unique oniscidean assemblages and we suggest that remnants of natural habitats within cities receive further attention in urban planning.

## Supplementary materials

Supplementary material 1 Geographical location and habitat characterization (urbanization intensity, soil and vegetation characteristics) of study sites in Buda, Hungary Data type: geographic location This is an open access article distributed under the terms of the Creative Commons Attribution License (CC BY 4.0), which permits unrestricted use, distribution, and reproduction in any medium, provided the original author and source are credited.Elisabeth Hornung, Andrea Kásler, Zsolt Tóth

Supplementary material 2 Species occurrence, richness and Average Rarity Index of study sites in Buda, Hungary Data type: species data This is an open access article distributed under the terms of the Creative Commons Attribution License (CC BY 4.0), which permits unrestricted use, distribution, and reproduction in any medium, provided the original author and source are credited.Elisabeth Hornung, Andrea Kásler, Zsolt Tóth
