# Supplementary material for: The role of urban forest patches in maintaining isopod diversity (Oniscidea)
Source: Zookeys. 2018 Dec 3;(801):371–88. doi: 10.3897/zookeys.801.22829 (PMC6288247; doi:10.3897/zookeys.801.22829)
Supplement: Supplementary material 1 — Geographical location and habitat characterization (urbanization intensity, soil and vegetation characteristics) of study sites in Buda, Hungary [file zookeys-801-371-s001.docx]

**Supplementary Table 1.** Geographical location and habitat characterization (urbanization intensity, soil and vegetation characteristics) of study sites in Buda, Hungary.

| **Site** | **Coordinates** | **UI** | **Soil properties** | | | | | **^†^Vegetation characteristics** | | | |
| --- | --- | --- | --- | --- | --- | --- | --- | --- | --- | --- | --- |
|  |  |  | **pH** | **K_A_** | **Salt** | **CaCO_3_** | **SOM** | **Dead wood** | **Litter cover** | **Litter depth** | **Canopy closure** |
| 1 | 47° 34' 25" N; 18° 56' 23" E | -1.15 | 6.16 | 59 | 0.01 | 0 | 7.11 | 3 | 3 | 3 | 3 |
| 2 | 47° 33' 49" N; 18° 59' 57" E | -1.47 | 7.46 | 43 | 0.01 | 11 | 4.82 | 1 | 1 | 1 | 1 |
| 3 | 47° 33' 50" N; 18° 59' 50" E | -2.11 | 7.04 | 82 | 0.01 | 1.9 | 8.5 | 2 | 2 | 3 | 3 |
| 4 | 47° 34' 06" N; 19° 01' 34" E | 2.87 | 7.8 | 49 | 0.01 | 18 | 5.78 | 2 | 2 | 2 | 2 |
| 5 | 47° 32' 07" N; 19° 01' 03" E | -0.11 | 6.82 | 58 | 0.01 | 0 | 8.5 | 3 | 3 | 3 | 2 |
| 6 | 47° 31' 33" N; 18° 56' 06" E | -1.56 | 7.4 | 64 | 0.01 | 6 | 8.5 | 3 | 3 | 3 | 3 |
| 7 | 47° 31' 14" N; 18° 56' 03" E | 2.73 | 7.73 | 57 | 0.01 | 13 | 7.28 | 2 | 1 | 1 | 1 |
| 8 | 47° 30' 50" N; 18° 56' 34" E | -1.16 | 8.04 | 45 | 0.01 | 18 | 3.45 | 2 | 3 | 3 | 3 |
| 9 | 47° 30' 53" N; 18° 56' 29" E | 0.27 | 7.51 | 48 | 0.01 | 18 | 3.79 | 2 | 1 | 1 | 1 |
| 10 | 47° 31' 45" N; 18° 57' 28" E | -1.75 | 7.69 | 57 | 0.01 | 5 | 6.49 | 2 | 3 | 3 | 2 |
| 11 | 47° 25' 56" N; 18° 59' 22" E | -2.58 | 7.57 | 56 | 0.01 | 20 | 7.67 | 2 | 3 | 3 | 3 |
| 12 | 47° 30' 57" N; 18° 58' 17" E | -1.36 | 7.32 | 73 | 0.01 | 32 | 8.5 | 2 | 3 | 3 | 2 |
| 13 | 47° 30' 47" N; 18° 58' 25" E | -1.03 | 7.29 | 54 | 0.02 | 20 | 8.5 | 3 | 3 | 3 | 3 |
| 14 | 47° 30' 55" N; 18° 58' 55" E | 0.74 | 6.87 | 54 | 0.03 | 0 | 7.73 | 3 | 3 | 3 | 3 |
| 15 | 47° 31' 45" N; 18° 57' 17" E | -1.64 | 6.28 | 64 | 0.05 | 0 | 8.5 | 2 | 2 | 2 | 3 |
| 16 | 47° 30' 19" N; 19° 00' 39" E | 2.24 | 7.62 | 55 | 0.02 | 30 | 6.72 | 2 | 1 | 3 | 2 |
| 17 | 47° 30' 18" N; 19° 00' 41" E | 2.07 | 6.92 | 59 | 0.01 | 37 | 3.75 | 1 | 1 | 1 | 1 |
| 18 | 47° 29' 12" N; 18° 59' 45" E | 2.37 | 7.45 | 43 | 0.01 | 22 | 3.99 | 2 | 1 | 2 | 2 |
| 19 | 47° 29' 10" N; 18° 58' 48" E | -1.35 | 6.76 | 73 | 0.02 | 0 | 8.5 | 3 | 3 | 2 | 3 |
| 20 | 47° 29' 08" N; 18° 58' 59" E | -0.31 | 7.27 | 45 | 0.05 | 3.2 | 6.45 | 2 | 1 | 1 | 1 |
| 21 | 47° 30' 05" N; 19° 01' 35" E | 5.56 | 7.5 | 56 | 0.05 | 16 | 6.48 | 2 | 1 | 1 | 1 |
| 22 | 47° 29' 21" N; 19° 02' 42" E | 0.44 | 7.54 | 62 | 0.01 | 38 | 8.5 | 2 | 1 | 2 | 2 |
| 23 | 47° 30' 11" N; 18° 57' 50" E | -1.71 | 7.36 | 58 | 0.01 | 2.7 | 8.5 | 3 | 3 | 3 | 3 |

Abbreviations – UI: urbanization index, K_A_: soil plasticity index according to Arany , SOM: soil organic matter. ^†^ Number 1,2 and 3 indicate the lowest, medium and the highest categories of vegetation features.
