# Supplementary material for: The role of urban forest patches in maintaining isopod diversity (Oniscidea)
Source: Zookeys. 2018 Dec 3;(801):371–88. doi: 10.3897/zookeys.801.22829 (PMC6288247; doi:10.3897/zookeys.801.22829)
Supplement: Supplementary material 2 — Species occurrence, richness and Average Rarity Index of study sites in Buda, Hungary [file zookeys-801-371-s002.docx]

**Supplementary Table 2.** Species occurrence, richness and Average Rarity Index of study sites in Buda, Hungary.

| **Site** | **Ort_pla** | **Pro_pol** | **Arm_vul** | **Cyl_con** | **Pla_hof** | **Por_sca** | **Por_spi** | **Por_pru** | **Por_col** | **Tra_nod** | **And_ros** | **Hap_men** | **Hyl_rip** | **Tri_pus** | **SR** | **ARI** |
| --- | --- | --- | --- | --- | --- | --- | --- | --- | --- | --- | --- | --- | --- | --- | --- | --- |
| 1 | X | X | 0 | 0 | 0 | 0 | 0 | 0 | 0 | 0 | 0 | 0 | X | 0 | 3 | 15.3 |
| 2 | X | 0 | X | 0 | 0 | X | 0 | 0 | 0 | 0 | X | X | X | 0 | 6 | 11.2 |
| 3 | X | X | 0 | 0 | X | 0 | 0 | 0 | 0 | 0 | 0 | 0 | 0 | 0 | 3 | 16 |
| 4 | 0 | 0 | X | 0 | 0 | 0 | 0 | X | 0 | X | 0 | 0 | X | X | 5 | 9.8 |
| 5 | 0 | 0 | 0 | 0 | 0 | 0 | 0 | 0 | X | 0 | 0 | 0 | 0 | 0 | 1 | 10 |
| 6 | X | X | 0 | 0 | 0 | 0 | 0 | 0 | 0 | 0 | 0 | 0 | 0 | 0 | 2 | 18 |
| 7 | 0 | 0 | 0 | 0 | 0 | X | 0 | 0 | 0 | 0 | 0 | 0 | 0 | 0 | 1 | 3 |
| 8 | X | X | 0 | 0 | 0 | 0 | 0 | 0 | 0 | 0 | 0 | 0 | 0 | 0 | 2 | 18 |
| 9 | 0 | 0 | X | 0 | 0 | X | 0 | 0 | 0 | 0 | 0 | 0 | 0 | 0 | 2 | 6 |
| 10 | X | X | X | 0 | 0 | 0 | 0 | 0 | 0 | 0 | 0 | 0 | 0 | 0 | 3 | 15 |
| 11 | 0 | 0 | X | 0 | 0 | 0 | 0 | 0 | 0 | 0 | 0 | 0 | 0 | 0 | 1 | 9 |
| 12 | 0 | 0 | 0 | 0 | X | 0 | 0 | 0 | 0 | 0 | 0 | 0 | 0 | 0 | 1 | 12 |
| 13 | X | X | 0 | 0 | 0 | 0 | 0 | 0 | 0 | 0 | 0 | 0 | 0 | 0 | 2 | 18 |
| 14 | X | X | X | X | 0 | X | 0 | 0 | 0 | 0 | 0 | 0 | 0 | 0 | 5 | 11.6 |
| 15 | X | X | 0 | 0 | 0 | 0 | X | 0 | 0 | 0 | 0 | 0 | 0 | 0 | 3 | 14.7 |
| 16 | 0 | 0 | X | X | X | 0 | X | 0 | X | 0 | 0 | 0 | 0 | X | 6 | 10.2 |
| 17 | 0 | 0 | X | 0 | 0 | X | X | 0 | X | 0 | 0 | 0 | 0 | 0 | 4 | 7.5 |
| 18 | 0 | 0 | X | X | X | X | 0 | X | 0 | 0 | 0 | 0 | X | 0 | 6 | 8.5 |
| 19 | X | X | 0 | 0 | 0 | 0 | 0 | 0 | 0 | 0 | 0 | 0 | 0 | 0 | 2 | 18 |
| 20 | 0 | 0 | X | 0 | 0 | X | 0 | X | 0 | 0 | 0 | 0 | 0 | 0 | 3 | 6.3 |
| 21 | 0 | 0 | X | 0 | 0 | X | 0 | 0 | 0 | 0 | 0 | 0 | 0 | 0 | 2 | 6 |
| 22 | 0 | 0 | X | 0 | X | 0 | 0 | 0 | X | 0 | 0 | 0 | 0 | 0 | 3 | 10.3 |
| 23 | X | 0 | X | 0 | 0 | 0 | 0 | 0 | 0 | 0 | 0 | 0 | 0 | X | 3 | 13.3 |

Abbreviations – Ort_pla: *Orthometopon planum*, Pro_pol: *Protracheoniscus politus*, Arm_vul: *Armadillidium vulgare*, Cyl_con: *Cylisticus convexus*, Pla_hof: *Platyarthrus hoffmannseggi*, Por_sca: *Porcellio scaber*, Por_spi: *Porcellio spinicornis*, Por_pru: *Porcellionides pruinosus*, Por_col: *Porcellium collicola*, Tra_nod: *Trachelipus nodulosus*, And_ros: *Androniscus roseus*, Hap_men: *Haplophthalmus mengii*, Hyl_rip: *Hyloniscus riparius*, Tri_pus: *Trichoniscus pusillus agg*; SR: species richness, ARI: Average Rarity Index
